# The Role of Outcome Response Rate in Planning Biosimilar Studies Using Different Evaluation Metrics

**DOI:** 10.3390/ph18020243

**Published:** 2025-02-12

**Authors:** Liyi Cen, Ramin Arani, Dejun Tang

**Affiliations:** Sandoz Inc., Princeton, NJ 08540, USA

**Keywords:** bioequivalence study, risk difference, risk ratio, equivalence margin conversion, overall response rate, biosimilar

## Abstract

**Background/Objectives:** Biosimilar studies use overall response rate to assess clinical similarity. Sample size and power depend on the equivalence margin, defined in either risk difference or risk ratio scale. This manuscript investigates how different evaluation metrics and varying response rates affect study power. **Methods:** Two numerical simulations are conducted. The first is designed to test in the risk difference scale, while the second tests in the risk ratio scale. Both simulations consider no difference between the biosimilar and reference product. Response rates vary from 0.1 to 0.9, and all scenarios are repeated 10,000 times. **Results:** The study shows inconsistent results in testing the equivalence of overall response rate across the risk difference and risk ratio scales, even when the hypotheses are mathematically equivalent. Consequently, the study is often under powered for testing in both scales. Additionally, study power is sensitive to outcome response rate deviation, with different directions of change in the two different evaluation metrics. **Conclusions:** Biosimilar study design should avoid the concept of converting equivalence margins between risk difference and risk ratio scales, assuming no change in study power. Careful strategies should be planned for estimating overall response rates for sample size assessments.

## 1. Introduction

In immuno-oncology biosimilar studies, the commonly used primary endpoint is the overall response rate within a specific time period to assess clinical similarity between a biosimilar and its reference product [1,2]. To demonstrate efficacy similarity, it is required to demonstrate that the clinical effect endpoints, for instance, the overall response rate, are equivalent for the proposed biosimilar and its reference product, measured by their corresponding predefined margins [1,3,4,5,6]. When the primary efficacy endpoint is chosen, a crucial aspect of designing a biosimilar study is to estimate the treatment effect and determine the equivalence margin.

The equivalence margin is closely related to the treatment effect size of the reference product. During the study design, the treatment effect size for the reference product is usually obtained by the lower limit of its 95% confidence interval, which is typically calculated through a meta-analysis of available data from historical studies [1,3]. Subsequently, the equivalence margin is set to preserve a certain percentage (typically 50%) of that effect size [3,4]. Based on the equivalence margin and the assumed treatment effect, the sample size and the power of the study can then be determined.

When using the overall response rate as the key efficacy endpoint, two commonly used evaluation metrics for comparing it between a biosimilar and its reference product are absolute risk difference and relative risk difference (or risk ratio) [7,8,9,10]. However, the choice of equivalence margin in these two metrics is subject to controversy, with no consensus reached within the scientific community or regulatory agencies. The scientific community has called for consensus on this matter [11]. Nevertheless, it is still common to design studies using both metrics to comply with requirements from various regulatory agencies, for example, in the several trastuzumab biosimilar clinical developments [9,12,13,14,15,16,17,18,19,20,21].

Once an equivalence margin is set for a trial, it is crucial to ensure the study has sufficient power to claim that the proposed biosimilar treatment is not different from the reference product. However, striking a balance between study power and a feasible sample size is a challenging decision for sponsors, especially for immuno-oncology studies [9,12]. As a key factor for the study power, outcome response rates are typically estimated based on historical data during the study design, but actual rates may differ from what are assumed in sample size assessments [7]. Consequently, the study power may vary depending on the actual response rate. The impact of this variation on study power is unclear, and it is also uncertain whether the same impact would occur if the statistical tests were conducted on risk difference or risk ratio scales.

There are two objectives for this manuscript. Both are related to the role of outcome response rate in designing a bioequivalence trial with two different evaluation metrics: risk difference and risk ratio. The first objective is to investigate the study power when the hypothesis testing is performed in the risk difference and risk ratio scale, respectively. We will examine the concordance of the two tests in different settings and explore the role of the overall response rate in this relationship. Second, we will investigate how the study power changes when the actual response rate differs from the assumed one during study design.

## 2. Results

### 2.1. Discordance of Equivalence Test in Risk Difference and Risk Ratio Scales

Table 1 presents the test results obtained in both risk difference and risk ratio scales when the study was originally designed to assess the risk difference. The equivalence margin for the risk ratio scale is derived from the equivalence margin of [−0.05, 0.05] in the risk difference scale, as outlined in Section 4.2. It is noted that the equivalence margin in the risk ratio scale varies for different response rates and is no longer symmetric. For each response rate, we analyzed the data in both risk difference and risk ratio scales and repeated the study 10,000 times. Table 1 reports the sample size per arm, converted equivalence margin in the risk ratio scale and the percentage of time that the study yields a positive result (i.e., null hypothesis is rejected) for the two tests in risk difference and risk ratio scales. Additionally, it reports the concordance between the two tests. The percentage of positive results for the test in the risk difference scale is very close to 80%, which aligns with the designed study power of 80%. Similarly, the percentage of positive results for the test in the risk ratio scale is also close to 80%, although it tends to be slightly lower compared to the test in the risk difference scale, particularly when the response rate is low. The discordance between the two tests ranges from 3.4% to 24.9%. A lower response rate is associated with a higher discordance. Consequently, the percentage of positive results for both tests is significantly lower than 80% when the response rate is low.

Table 2 presents the test results obtained in both risk difference and risk ratio scales when the study was originally designed to assess the risk ratio. The equivalence margin for the risk difference scale is derived from the equivalence margin of [$\frac{1}{1.2}$, 1.2] in the risk ratio scale, as outlined in Section 4.2. It also varies for different response rates and is no longer symmetric. The percentage of positive results for the test in the risk ratio scale is very close to 80%, which aligns with the designed study power of 80%. Similarly, the percentage of positive results for the test in the risk difference scale is also close to 80%. The discordance between the two tests is around 10% in all scenarios. Consequently, the percentage of positive results for both tests is a few percent lower than 80%.

**Figure 1:**
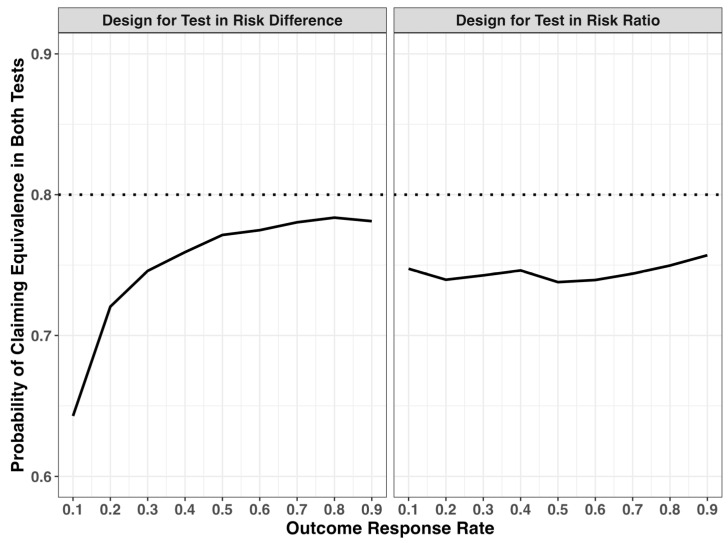
Percentage of positive results for both tests in risk difference and risk ratio scales when (**left**) the study is originally designed to test in risk difference and (**right**) the study is originally designed to test in risk ratio.

Figure 1 illustrates the comparison between the outcome response rate and the percentage of positive results for both tests. When the study is initially designed to test in the risk ratio scale (Figure 1, right panel), the percentage of positive results remains relatively consistent around 75%. However, when the study is initially designed to test in the risk difference scale (Figure 1, left panel), the percentage of positive results varies significantly with the response rate. Notably, a significantly low percentage of positive results for both tests is observed when the response rate is low in the latter case.

### 2.2. Sensitivity of Study Power When the Outcome Response Rate Deviates from the Assumed Level During Study Design

**Figure 2:**
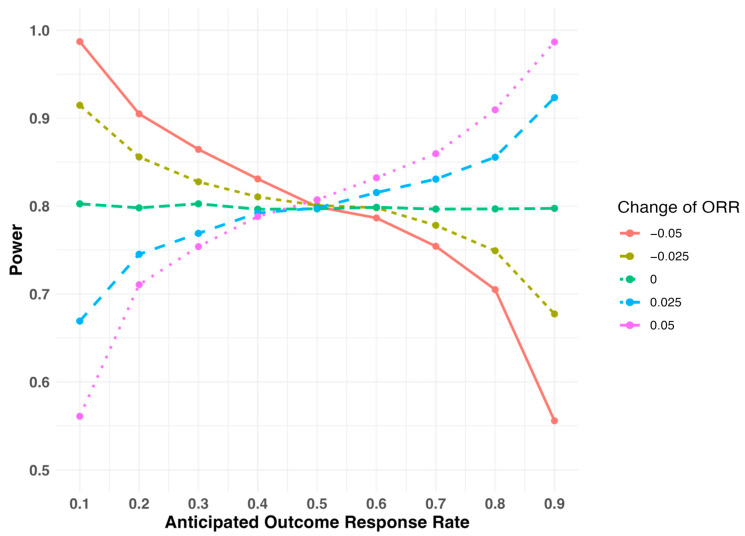
Study power with respect to the assumed outcome response rate (ORR) at different levels of deviations when the study is initially designed to test in the risk difference scale.

When the study is initially designed to test for the risk difference, the study power with respect to the assumed outcome response rate during the study design is illustrated at different levels of deviations in Figure 2. If the outcome response rate is overestimated, meaning the actual outcome response rate is lower than the originally assumed rate (e.g., the red line in Figure 2 represents the scenario in which the actual outcome response rate is 0.05 lower than the assumed level), the study power is larger than the planned 80% level when the outcome response rate is below 0.5. Conversely, the study power is smaller than the planned 80% level when the outcome response rate is above 0.5. If the outcome response rate is underestimated, meaning the actual outcome response rate is higher than the assumed rate (e.g., the purple line in Figure 2 represents the scenario in which the actual outcome response rate is 0.05 higher than the assumed level), the study power is smaller than the planned 80% level when the outcome response rate is less than 0.5. The study power is larger than the planned 80% level if the outcome response rate is greater than 0.5. Furthermore, the study power remains relatively stable regardless of the change from the assumed response rate if the outcome response rate is close to 0.5. For example, with the assumed response rate of 0.5, the study power is 81% or 80.4% if the response rate reduces to 0.45 or increases to 0.55, respectively (Table 3, Study Design I). However, if the outcome response rate is either low (close to 0) or high (close to 1), the study power can change quite a bit, even for a small change in the response rate. For example, with the assumed response rate of 0.2, the study power increases to 90.2% if the response rate reduces to 0.15 (a change of −0.05). The study power reduces to only 69.9% if the response rate increases to 0.25 (a change of 0.05).

**Figure 3:**
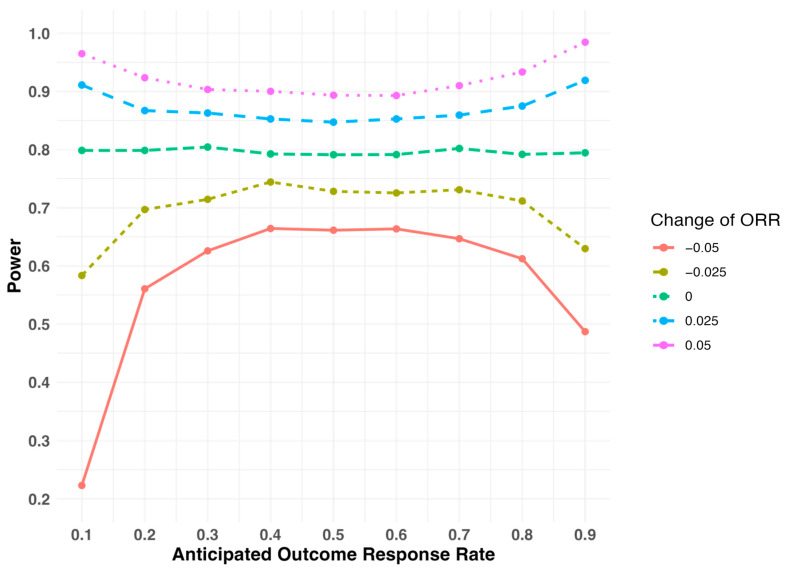
Study power with respect to the assumed outcome response rate (ORR) at different levels of deviations when the study is initially designed to test in the risk ratio scale.

When the study is designed to test for the risk ratio, the relationship between study power and the change in outcome response rate is quite different (Figure 3). The study power increases with an increase in the outcome response rate and decreases with a decrease in the outcome response rate, regardless of its absolute level. Moreover, the change in study power is larger when the outcome response rate is either low (close to 0) or high (close to 1) compared to when it is close to 0.5. For example, with the assumed response rate of 0.5, the study power reduces to 66.1% if the response rate reduces to 0.45 by 0.05. With the assumed response rate of 0.2, the study power reduces to 56.1% if the response rate reduces to 0.15 by 0.05 (Table 3, Study Design II).

## 3. Discussions

In this manuscript, we demonstrate that the study lacks sufficient power to detect equivalence using both metrics if it was initially designed to test only in one metric due to the discordance of the two test results. Additionally, the study power can be sensitive to the outcome response rate when it deviates from the assumed level. Furthermore, the direction of change in power can differ depending on whether the risk difference or risk ratio metric is used.

We employed two study designs to compare the study power evaluated in the risk difference and risk ratio scales as well as the concordance of the test results between the two. When using the equivalence margins between the two metrics that maintain the same hypotheses, we discovered significant discordance between the two tests. The magnitude of this discordance depends on the outcome response rate and the original evaluation metric used in designing the study. Consequently, if a study was originally designed to have sufficient power in one test only, it may be severely underpowered to claim equivalence in both tests due to this discordance. This finding has significant implications for designing the study. It is crucial to recognize that, if a study concludes equivalence using one metric, it does not necessarily imply the same conclusion using the other metric, even if the equivalence margin is calculated to ensure the same hypotheses are tested between the two metrics. Therefore, when designing a biosimilar study, it is essential to avoid the concept of conversion in the equivalence margin and assume that the study power will remain the same.

Instead, if the sample size is calculated to ensure sufficient power for claiming equivalence in one test, it must be increased to achieve the same level of power for both tests. The extent of this sample size increase depends on which test the study was originally powered for and, possibly, the outcome response rate. If we begin with a study design that ensures sufficient power for testing in the risk ratio scale, the percentage increase in sample size remains relatively constant regardless of the outcome response rate. In our numerical simulations, we found that a 10% increase in sample size ensures the study has at least 80% power to claim equivalence in both tests. However, if the study was originally designed to ensure sufficient power for testing in the risk difference scale, the percentage increase in sample size also depends on the outcome response rate. A lower outcome response rate necessitates a larger sample size increase. In practical terms, it may be more operationally convenient to calculate the sample size in the risk ratio scale and then increase it by a specific percentage to ensure sufficient power in both tests, assuming that the equivalence margin defined by the mathematically converted upper and lower bound in the risk difference scale is clinically acceptable.

Sponsors may also consider the impact on the study duration and cost with the increase in sample size. Moore et al. [22] reported that the median enrollment for 24 biosimilar phase 3 trials conducted for products approved between January 2010 and October 2019 was 538 participants. The median trial completion time of these studies was 26 months, with a median cost of $27.6 million. Assuming a proportional relationship between the sample size, cost and time, a 10% increase in sample size would lead to an estimated cost increase of $2.76 million and extend the trial duration by roughly 2.6 months. However, it is important to note that the actual impact may vary depending on different factors, such as therapeutic indication, recruitment rate and operational cost.

The simulation results presented in this manuscript are from the parallel design. The same principle would apply if other types of designs (e.g., crossover or hybrid parallel-crossover design) need to use both metrics to evaluate the similarity. It is recommended to consider the equivalence margin carefully in different metrics instead of simply converting the margin from one scale into the other.

During clinical study design, it is common to assume a fixed outcome response rate to determine the sample size. However, the actual response rate can vary. Macaya et al. [11] performed a literature review from 2010 to 2015 and identified nine non-inferiority trials comparing new-generation to second-generation stents. They reported that the observed event rate was lower than expected in all but one study. In some trials, the difference was substantial. As a result, only four of the nine trials consistently demonstrated non-inferiority using the relative risk metric as compared to the original margin defined in rate difference in those studies. The two numerical studies in this manuscript illustrated how the response rate affects study power when using different evaluation metrics. When the outcome response rate is below 0.5, the study power calculated using equivalence margins defined in the two metrics changes in opposite directions with the response rate. An increase in the response rate leads to reduced power for the test in risk difference and increased power for the test in risk ratio. In this case, a more precise estimate of the outcome response rate is crucial to maintain the study power as intended. On the other hand, when the outcome response rate is above 0.5, the study power for both metrics changes in the same direction with the change in outcome response rate. An increase in the response rate leads to an increase in the study power. In this situation, it is better to be conservative, assuming a lower response rate for sample size assessments. Li et al. [7] suggested that different scenarios of event rates should be considered at the design stage to ensure adequate power for the chosen margin in non-inferiority trials. Our finding is also consistent with the recommendation for equivalence trials, particularly for trials using both RR and RD metrics as required by different health authorities.

## 4. Methods

### 4.1. Equivalence Test of Outcome Response Rate

We first review how the hypothesis test is performed using the risk difference metric. Let $p_{t}$ and $p_{r}$ denote the outcome response rate in the biosimilar arm and the reference arm, respectively. When the risk difference evaluation metric is used, the null ${(H}_{0})$ and alternative ${(H}_{1})$ hypotheses for equivalence studies are as follows:

$$H_{0}:\;p_{t}-p_{r}\leq \delta_{L}\;\mathrm{or}\;p_{t}-p_{r}\geq \delta_{U}$$

$$H_{1}:\;\delta_{L}<p_{t}-p_{r}<\delta_{U}$$

where $\left[\delta_{L},\;\delta_{U}\right]$ is a pre-specified equivalence margin. In biosimilar development, the target risk difference is zero in most cases so that the equivalence margin is often symmetric around zero, i.e., $\delta_{U}=-\delta_{L}$. If we set $\delta_{U}=\delta$, the equivalence margin is [−δ,δ].

The equivalence test is usually performed using two one-sided tests [23]. For the left-sided test, the null and alternative hypotheses are

$$H_{0,L}:\;p_{t}-p_{r}\leq -\delta \;\mathrm{vs}.\;H_{1,L}:\;p_{t}-p_{r}>-\delta$$

For the right-sided test, the null and alternative hypotheses are

$$H_{0,U}:\;p_{t}-p_{r}\geq \delta \;\mathrm{vs}.\;H_{1,U}:\;p_{t}-p_{r}<\delta$$

The equivalence test can be carried out similarly using the risk ratio evaluation metric. The null and alternative hypotheses are

$$H_{0}:\;\frac{p_{t}}{p_{r}}\leq \lambda_{L}\;or\;\frac{p_{t}}{p_{r}}\geq \lambda_{U}$$

$$H_{1}:\;\lambda_{L}<\frac{p_{t}}{p_{r}}<\lambda_{U}$$

where $\left[\lambda_{L},\;\lambda_{U}\right]$ is the equivalence margin defined in the risk ratio scale. The target risk ratio is usually 1 so that, for a symmetric equivalence margin, we can set $\lambda_{L}=\frac{1}{\lambda_{U}}$ (assume that $\lambda_{U}>1$). If we set $\lambda_{U}=\lambda$, the symmetric equivalence margin becomes $\left[\frac{1}{\lambda },\lambda \right]$.

Similar to the test in risk difference scale, the test in risk ratio scale can also be carried out using two one-sided tests. For the left-sided test, the null and alternative hypotheses are

$$H_{0,L}:\;\frac{p_{t}}{p_{r}}\leq \frac{1}{\lambda }\;\mathrm{vs}.\;H_{1,L}:\;\frac{p_{t}}{p_{r}}>\frac{1}{\lambda }$$

For the right-sided test, the null and alternative hypotheses are

$$H_{0,U}:\;\frac{p_{t}}{p_{r}}\geq \lambda \;\mathrm{vs}.\;H_{1,U}:\;\frac{p_{t}}{p_{r}}<\lambda$$

### 4.2. Equivalence Margin Conversion Between Risk Difference and Risk Ratio Scale

Biosimilar studies sometimes aim to establish equivalence in both risk difference and risk ratio scales to meet requirements from different regulatory agencies. This would require the specification of equivalence margin for the risk difference and risk ratio separately in one study. Under certain conditions, the null and alternative hypotheses in the two one-sided tests in the risk difference scale are equivalent to the two one-sided tests in the risk ratio scale as follows.

Assume the overall response rate for the reference product is p, and we design the study based on the risk difference and pre-specify [−δ, δ] as the equivalence margin for the test in risk difference scale. The alternative hypothesis states that the overall response rate for the biosimilar product falls within [p−δ, p+δ], which is equivalent to saying that the risk ratio between the two products falls within $\left[\frac{p-\delta }{p},\;\frac{p+\delta }{p}\right]$. Therefore, if we set the equivalence margin for the risk ratio as $[1-\frac{\delta }{p},\;1+\frac{\delta }{p}]$, the two hypothesis tests in the risk difference and risk ratio scale are equivalent. However, note that the equivalence margin for the risk ratio is not symmetric anymore. Although the hypothesis tests are mathematically equivalent in the two scales, the power of the equivalent test for the risk ratio will be different since the margin is asymmetric.

Similarly, we can perform the test using the risk ratio metric with a pre-specified equivalence margin for the risk ratio as $\left[\frac{1}{\lambda },\lambda \right]$ (assume λ>1). The alternative hypothesis states that the overall response rate for the biosimilar product falls within $\left[\frac{p}{\lambda },\;p\lambda \right]$, which is equivalent to saying that the risk difference between the two products falls within $\left[\frac{p}{\lambda }-p,\;p\lambda -p\right]$. Therefore, if we set the equivalence margin for the risk difference as $\left[\left(\frac{1}{\lambda }-1\right)p,(\lambda -1)p\right]$, the two hypothesis tests in the risk difference and risk ratio scale are again equivalent. Again, the equivalence margin for the risk difference is not symmetric anymore. Due to the same reason mentioned above, the power of the test for the risk difference will be different due to the asymmetric margin.

We will use this as our basis for relating the equivalence margin from one scale to the other in the numerical simulation studies.

### 4.3. Simulation Study Setup

We conduct two simulation studies to investigate the two study objectives. Table 4 outlines the design of the two studies. The first study is designed to perform an equivalence test using the risk difference metric, while the second study is designed to perform the test using the risk ratio metric. We consider a series of response rates for the reference product between 0.1 and 0.9. We consider the case when there is no difference in the overall response rate between the biosimilar and reference product (i.e., the alternative hypothesis is true).

For the first study, we set the equivalence margin as [−0.05, 0.05] on the risk difference scale. For the second study, we set the equivalence margin as [$\frac{1}{1.2}$, 1.2] on the risk ratio scale. Based on the assumed response rates and equivalence margins, we calculate the sample size to ensure that the test has 80% power in the originally selected scale (risk difference scale in study I and risk ratio scale in study II) with a type I error rate of 0.05. To assess the study power in scales other than the original design, we use the equivalence margin defined by the mathematically converted lower and upper bound suggested in the previous section.

Here is the summary of simulation steps: (1) simulate binary outcomes with predetermined response rates and sample sizes for both treatment arms; (2) estimate the risk difference, risk ratio, and corresponding 95% confidence intervals from the simulated data; (3) compare the 95% confidence interval with the equivalence margin to determine if the equivalence is established in the risk difference and risk ratio scale; (4) compare the conclusions from the two tests conducted in the two different scales. The R program used for the simulation is provided in the Supplementary Materials.

Additionally, we evaluate the study power when the observed response rate differs from the assumed response rate during study design. We conduct two tests in both the risk difference and risk ratio scales, varying the response rate deviation among four levels: −0.05, −0.025, 0.025 and 0.05. In both studies, all scenarios are repeated 10,000 times.

## 5. Conclusions

In summary, when designing a biosimilar study, it is essential to avoid the concept of conversion in the equivalence margin between the risk difference and risk ratio scales and assume that the study power remains the same. Additionally, a careful strategy should be considered for estimating the overall response rate for the purpose of sample size assessments.

## Figures and Tables

**Table 1:** Comparison of equivalence test results in risk difference and risk ratio scales in study design I. The sample size is selected such that the study power is 80% when testing in the risk difference scale with type I error rate of 0.05. All scenarios were repeated 10,000 times.

Response Rate	Sample Size Per Arm	RR Margin ^1,2^		Percent Time with Positive Test Result					
				RD Test	RR Test	Both Test	RD Test Only	RR Test Only	Discordance ^3^
0.1	757	0.50	1.50	79.7%	73.7%	64.3%	15.5%	9.4%	24.9%
0.2	1345	0.75	1.25	79.8%	78.1%	72.1%	7.8%	6.1%	13.8%
0.3	1766	0.83	1.17	79.4%	79.1%	74.6%	4.8%	4.5%	9.3%
0.4	2018	0.88	1.13	79.7%	79.1%	75.9%	3.8%	3.2%	7.0%
0.5	2102	0.90	1.10	80.1%	79.8%	77.1%	3.0%	2.6%	5.6%
0.6	2018	0.92	1.08	80.0%	79.8%	77.5%	2.6%	2.3%	4.8%
0.7	1766	0.93	1.07	79.9%	79.8%	78.0%	1.9%	1.8%	3.6%
0.8	1345	0.94	1.06	80.2%	80.2%	78.4%	1.8%	1.8%	3.6%
0.9	757	0.94	1.06	80.0%	79.7%	78.1%	1.8%	1.6%	3.4%

^1^ RD: risk difference; RR: risk ratio. ^2^ The equivalence margin for risk ratio is calculated from the equivalence margin for risk difference using the formula in Section 4.2. ^3^ The discordance is the sum of the “RD test only” and “RR test only” columns.

**Table 2:** Comparison of equivalence test results in risk difference and risk ratio scales in study design II. The sample size is selected such that the study power is 80% when testing in the risk ratio scale with type I error rate of 0.05. All scenarios were repeated 10,000 times.

Overall Response Rate	Sample Size Per Arm	RD Margin ^1,2^		Percent Time with Positive Test Result					
				RD Test	RR Test	Both Test	RD Test Only	RR Test Only	Discordance ^3^
0.1	5712	−0.02	0.02	79.5%	80.5%	74.7%	4.7%	5.8%	10.5%
0.2	2530	−0.03	0.04	78.3%	79.7%	74.0%	4.4%	5.8%	10.1%
0.3	1483	−0.05	0.06	79.1%	79.8%	74.3%	4.9%	5.5%	10.4%
0.4	954	−0.07	0.08	79.1%	80.3%	74.6%	4.5%	5.7%	10.2%
0.5	628	−0.08	0.10	78.8%	79.6%	73.8%	5.0%	5.8%	10.9%
0.6	418	−0.10	0.12	79.0%	79.4%	73.9%	5.1%	5.5%	10.6%
0.7	274	−0.12	0.14	79.0%	79.9%	74.4%	4.6%	5.5%	10.1%
0.8	160	−0.13	0.16	80.2%	80.0%	75.0%	5.2%	5.0%	10.2%
0.9	72	−0.15	0.18	80.7%	80.7%	75.7%	5.0%	5.0%	10.0%

^1^ RR: risk difference; RD: risk ratio. ^2^ The equivalence margin for risk difference is calculated from the equivalence margin for risk ratio using the formula in Section 4.2. ^3^ The discordance is the sum of the “RD test only” and “RR test only” columns.

**Table 3:** Power of study when the observed response rate is deviated from the expected response rate by 0.05.

Assumed Response Rate	Study Design I		Study Design II	
	Actual Response Rate Is 0.05 Smaller Than Expected	Actual Response Rate Is 0.05 Larger Than Expected	Actual Response Rate Is 0.05 Smaller Than Expected	Actual Response Rate Is 0.05 Larger Than Expected
0.1	98.8%	55.4%	22.3%	96.5%
0.2	90.2%	69.9%	56.1%	92.4%
0.3	85.8%	75.5%	62.6%	90.3%
0.4	83.8%	77.9%	66.4%	90.0%
0.5	81.0%	80.4%	66.1%	89.4%
0.6	78.7%	83.2%	66.4%	89.3%
0.7	75.1%	86.1%	64.7%	91.0%
0.8	70.5%	90.8%	61.2%	93.3%
0.9	55.6%	98.6%	48.7%	98.5%

**Table 4:** Summary of study design.

	Study Design I	Study Design II
Metric of evaluation	Risk Difference	Risk Ratio
Reference response rate	10% to 90%	10% to 90%
Expected difference between treatments	RD = 0	RR = 1
Equivalence margin	[−0.05, 0.05]	$[\frac{1}{1.2}$, 1.2]
Type I error	0.05	0.05
Study power	0.8	0.8

## Data Availability

Data is contained within the article or Supplementary Materials.

## Supplementary Materials

The following supporting information can be downloaded at: https://www.mdpi.com/article/10.3390/ph18020243/s1. The supplementary section includes the R program used for the simulation.
