# Supplementary material for: The Role of Outcome Response Rate in Planning Biosimilar Studies Using Different Evaluation Metrics
Source: Pharmaceuticals (Basel). 2025 Feb 12;18(2):243. doi: 10.3390/ph18020243 (PMC11860014; doi:10.3390/ph18020243)
Supplement: Supplementary file 1 [file pharmaceuticals-18-00243-s001.zip › Supplemental material.docx]

**R Code for the simulation study**

library(ggplot2)

library(parallel)

rd_rr_eqm_simple <- function(n_arm, mu.b, mu.r, rrm.l, rrm.u, rdm.l, rdm.u, alpha) {

# Simulate data

y.b <- rbinom(n_arm, 1, mu.b)

y.r <- rbinom(n_arm, 1, mu.r)

# Calculate probabilities

p1 <- mean(y.b)

p2 <- mean(y.r)

# Risk Difference (RD)

rd <- p2 - p1

se_rd <- sqrt((p1 * (1 - p1) / n_arm) + (p2 * (1 - p2) / n_arm))

ci_rd <- c(rd - qnorm(1 - alpha / 2) * se_rd, rd + qnorm(1 - alpha / 2) * se_rd)

rd_pass <- ci_rd[1] > rdm.l & ci_rd[2] < rdm.u

# Relative Risk (RR)

rr <- ifelse(p1 > 0, p2 / p1, Inf) # Handle division by zero for p1

if (p1 > 0 && p2 > 0) {

se_ln_rr <- sqrt((1 - p1) / (n_arm * p1) + (1 - p2) / (n_arm * p2))

ci_rr <- exp(c(log(rr) - qnorm(1 - alpha / 2) * se_ln_rr,

log(rr) + qnorm(1 - alpha / 2) * se_ln_rr))

} else {

ci_rr <- c(NA, NA) # Undefined confidence interval

}

rr_pass <- ifelse(p1 > 0, ci_rr[1] > rrm.l & ci_rr[2] < rrm.u, FALSE)

# Concordance

pass_both <- rd_pass & rr_pass

pass_rd <- rd_pass & !rr_pass

pass_rr <- rr_pass & !rd_pass

discordance <- rd_pass != rr_pass

return(c(rd_pass = rd_pass, rr_pass=rr_pass, both = pass_both, rd_only = pass_rd, rr_only = pass_rr,

discordance = discordance))

}

##############################

### Table 1

##############################

p.list.rd <- seq(0.1, 0.9, 0.1)

n_arm.list.rd <- c(757, 1345, 1766, 2018, 2102, 2018, 1766, 1345, 757)

set.seed(20210511)

summary.rd <- NULL

for (i in 1:length(p.list.rd)){

p <- p.list.rd[i]

n_arm <- n_arm.list.rd[i]

delta <- 0.05

mu.b <- p

mu.r <- p

rrm.l <- 1 - delta/p

rrm.u <- 1 + delta/p

rdm.l <- -1 * delta

rdm.u <- delta

alpha <- 0.05

sim_rd_rr_eqm_simple <- replicate(10000, rd_rr_eqm_simple(n_arm=n_arm, mu.b=mu.b, mu.r=mu.r, rrm.l=rrm.l, rrm.u=rrm.u,

rdm.l=rdm.l, rdm.u=rdm.u, alpha=alpha))

out <- c(p, n_arm, rdm.l, rdm.u, rrm.l, rrm.u, rowMeans(sim_rd_rr_eqm_simple))

summary.rd <- rbind(summary.rd,out)

}

colnames(summary.rd) <- c("p", "n_arm", "rdm.l", "rdm.u", "rrm.l", "rrm.u",

"rd_pass", "rr_pass", "both_pass", "rd_only", "rr_only", "discordance")

row.names(summary.rd) <- NULL

print(summary.rd)

##############################

### Table 2

##############################

p.list.rr <- seq(0.1, 0.9, 0.1)

n_arm.list.rr <- c(5712, 2530, 1483, 954, 628, 418, 274, 160, 72)

set.seed(20210511)

summary.rr <- NULL

for (i in 1:length(p.list.rr)){

p <- p.list.rr[i]

n_arm <- n_arm.list.rr[i]

lambda <- 1.2

mu.b <- p

mu.r <- p

rrm.l <- 1/lambda

rrm.u <- lambda

rdm.l <- p/lambda - p

rdm.u <- p*lambda - p

alpha <- 0.05

sim_rd_rr_eqm_simple <- replicate(10000, rd_rr_eqm_simple(n_arm=n_arm, mu.b=mu.b, mu.r=mu.r, rrm.l=rrm.l, rrm.u=rrm.u,

rdm.l=rdm.l, rdm.u=rdm.u, alpha=alpha))

out <- c(p, n_arm, rrm.l, rrm.u, rdm.l, rdm.u, rowMeans(sim_rd_rr_eqm_simple))

summary.rr <- rbind(summary.rr,out)

}

colnames(summary.rr) <- c("p", "n_arm", "rrm.l", "rrm.u", "rdm.l", "rdm.u",

"rd_pass", "rr_pass", "both_pass", "rd_only", "rr_only", "discordance")

row.names(summary.rr) <- NULL

print(summary.rr)

##############################

### Figure 1

##############################

summary.rd1 <- as.data.frame(summary.rd)

summary.rd1$scenario <- "Design for Test in Risk Difference"

summary.rr1 <- as.data.frame(summary.rr)

summary.rr1$scenario <- "Design for Test in Risk Ratio"

summary.all <- rbind(summary.rd1, summary.rr1)

figure1 <- ggplot(summary.all, aes(x = p, y = both_pass)) +

geom_line(linewidth = 1) +

theme_bw() +

theme(

legend.title = element_text(size = 12),

axis.text = element_text(size = 12, face = "bold"),

axis.title = element_text(size = 14, face = "bold"),

strip.text = element_text(size = 12, face = "bold")

) +

scale_x_continuous(

breaks = seq(0.1, 0.9, 0.1),

limits = c(0.1, 0.9)

) +

scale_y_continuous(

breaks = seq(0.6, 0.9, 0.1),

limits = c(0.6, 0.9)

) +

xlab("Outcome Response Rate") +

ylab("Probability of Claiming Equivalence in Both Tests") +

geom_hline(

yintercept = 0.8,

linetype = "dotted",

color = "black",

linewidth = 1

) +

facet_grid(cols = vars(scenario))

##############################

### Figure 2

##############################

power_rd_do_once <- function(n_arm, mu.b, mu.r, rdm.l, rdm.u, alpha) {

# Simulate data

y.b <- rbinom(n_arm, 1, mu.b)

y.r <- rbinom(n_arm, 1, mu.r)

# Calculate probabilities

p1 <- mean(y.b)

p2 <- mean(y.r)

# Risk Difference (RD)

rd <- p2 - p1

se_rd <- sqrt((p1 * (1 - p1) / n_arm) + (p2 * (1 - p2) / n_arm))

ci_rd <- c(rd - qnorm(1 - alpha / 2) * se_rd, rd + qnorm(1 - alpha / 2) * se_rd)

rd_pass <- ci_rd[1] > rdm.l & ci_rd[2] < rdm.u

return(rd_pass)

}

power_rd_parallel <- function(n_arm, mu.b, mu.r, rdm.l, rdm.u, alpha, n_sim) {

cl <- makeCluster(detectCores() - 1) # Use available cores minus 1

# Export the function and variables explicitly to the cluster

clusterExport(cl, varlist = c("power_rd_do_once"))

clusterExport(cl, varlist = c("n_arm", "mu.b", "mu.r", "rdm.l", "rdm.u", "alpha"), envir = environment())

results <- parSapply(cl, 1:n_sim, function(x) power_rd_do_once(n_arm, mu.b, mu.r, rdm.l, rdm.u, alpha))

stopCluster(cl)

mean(results)

}

p.list.rd <- seq(0.1, 0.9, 0.1)

n_arm.list.rd <- c(757, 1345, 1766, 2018, 2102, 2018, 1766, 1345, 757)

pct.list.rd <- seq(-0.05, 0.05, 0.025)

# Preallocate a results matrix

results_rd <- NULL

set.seed(588437)

# Nested loop

for (i in 1:length(p.list.rd)) {

for (j in 1:length(pct.list.rd)) {

# Adjust n_arm based on percentage change

p.tmp <- p.list.rd[i] + pct.list.rd[j]

# Calculate power

power.tmp <- power_rd_parallel(n_arm.list.rd[i],

p.tmp,

p.tmp,

-0.05, 0.05, 0.05,

10000)

results_rd <- rbind(results_rd, c(p.list.rd[i], pct.list.rd[j], power.tmp))

}

}

results_rd_df <- as.data.frame(results_rd)

colnames(results_rd_df) <- c("p.list", "pct.list", "power")

figure2 <- ggplot(results_rd_df, aes(x = p.list, y = power, color = as.factor(pct.list), linetype = as.factor(pct.list), group = pct.list)) +

geom_line(linewidth = 1) +

geom_point(size = 2) +

labs(

x = "Anticipated Outcome Response Rate",

y = "Power",

color = "Change of ORR",

linetype = "Change of ORR"

) +

scale_x_continuous(

breaks = seq(0.1, 0.9, 0.1),

limits = c(0.1, 0.9)

) +

scale_y_continuous(

breaks = seq(0.5, 1, 0.1),

limits = c(0.5, 1)

) +

theme_minimal() +

theme(

legend.title = element_text(size = 12),

axis.text = element_text(size = 12, face = "bold"),

axis.title = element_text(size = 14, face = "bold"),

legend.position = "right"

)

##############################

### Figure 3

##############################

power_rr_do_once <- function(n_arm, mu.b, mu.r, rrm.l, rrm.u, alpha) {

# Simulate data

y.b <- rbinom(n_arm, 1, mu.b)

y.r <- rbinom(n_arm, 1, mu.r)

# Calculate probabilities

p1 <- mean(y.b)

p2 <- mean(y.r)

rr <- ifelse(p1 > 0, p2 / p1, Inf) # Handle division by zero for p1

if (p1 > 0 && p2 > 0) {

se_ln_rr <- sqrt((1 - p1) / (n_arm * p1) + (1 - p2) / (n_arm * p2))

ci_rr <- exp(c(log(rr) - qnorm(1 - alpha / 2) * se_ln_rr,

log(rr) + qnorm(1 - alpha / 2) * se_ln_rr))

} else {

ci_rr <- c(NA, NA) # Undefined confidence interval

}

rr_pass <- ifelse(p1 > 0, ci_rr[1] > rrm.l & ci_rr[2] < rrm.u, FALSE)

return(rr_pass)

}

power_rr_parallel <- function(n_arm, mu.b, mu.r, rrm.l, rrm.u, alpha, n_sim = 10000) {

cl <- makeCluster(detectCores() - 1) # Use available cores minus 1

clusterExport(cl, varlist = c("power_rr_do_once"))

clusterExport(cl, varlist = c("n_arm", "mu.b", "mu.r", "rrm.l", "rrm.u", "alpha"), envir = environment())

results <- parSapply(cl, 1:n_sim, function(x) power_rr_do_once(n_arm, mu.b, mu.r, rrm.l, rrm.u, alpha))

stopCluster(cl)

mean(results)

}

p.list.rr <- seq(0.1, 0.9, 0.1)

n_arm.list.rr <- c(5712, 2530, 1483, 954, 628, 418, 274, 160, 72)

pct.list.rr <- seq(-0.05, 0.05, 0.025)

# Preallocate a results matrix

results.rr <- NULL

set.seed(8979467)

# Nested loop

for (i in 1:length(p.list.rr)) {

for (j in 1:length(pct.list.rr)) {

# Adjust n_arm based on percentage change

p.tmp <- p.list.rr[i] + pct.list.rr[j]

# Calculate power

power.tmp <- power_rr_parallel(n_arm.list.rr[i],

p.tmp,

p.tmp,

1/1.2, 1.2, 0.05,

10000)

results.rr <- rbind(results.rr, c(p.list.rr[i], pct.list.rr[j], power.tmp))

}

}

results_rr_df <- as.data.frame(results.rr)

colnames(results_rr_df) <- c("p.list", "pct.list", "power")

figure3 <- ggplot(results_rr_df, aes(x = p.list, y = power, color = as.factor(pct.list), linetype = as.factor(pct.list), group = pct.list)) +

geom_line(linewidth = 1) +

geom_point(size = 2) +

labs(

x = "Anticipated Outcome Response Rate",

y = "Power",

color = "Change of ORR",

linetype = "Change of ORR"

) +

scale_x_continuous(

breaks = seq(0.1, 0.9, 0.1),

limits = c(0.1, 0.9)

) +

scale_y_continuous(

breaks = seq(0.2, 1, 0.1),

limits = c(0.2, 1)

) +

theme_minimal() +

theme(

legend.title = element_text(size = 12),

axis.text = element_text(size = 12, face = "bold"),

axis.title = element_text(size = 14, face = "bold"),

legend.position = "right"

)
